# Clinicopathological and Immunohistochemical Correlation of Stromal CD10 Expression in Invasive Breast Carcinoma: A Prospective Observational Study

**DOI:** 10.22034/ijp.2026.2087169.3640

**Published:** 2026-08-01

**Authors:** Carlin Kirthika. P, Jawahar Ramasamy, Vigneswara Moorthi, Prabhu, Siva Kaliyamoorthy, Varadharajaperumal Radhakrishnan

**Affiliations:** 1 Department of Pathology, Aarupadai Veedu Medical College and Hospital, Vinayaka Missions Research Foundation, (Deemed to be University), Puducherry, India; 2 Department of General Surgery, Aarupadai Veedu Medical College and Hospital, Vinayaka Missions Research Foundation, (Deemed to be University), Puducherry, India

**Keywords:** CD10, Breast carcinoma, Immunohistochemistry, Stromal marker

## Abstract

**Background & Objective:**

Breast cancer is a major global health concern, with tumor heterogeneity and tumor microenvironment (TME) dynamics limiting the predictive value of conventional prognostic markers such as age, tumor size, grade, nodal status, lymphovascular invasion (LVI), and ER/PR/HER2 status. Stromal CD10, a zinc-dependent metalloproteinase expressed by fibroblasts and myoepithelial cells, promotes tumor invasion through extracellular matrix remodeling and is associated with aggressive tumor features. This study evaluates stromal CD10 expression in invasive breast carcinoma and its correlation with clinicopathological and immunohistochemical parameters.

**Methods:**

A prospective study of 73 modified radical mastectomy specimens of invasive ductal carcinoma was conducted between April 2024 and December 2025. Histopathological diagnosis and grading were confirmed using H&E staining. Immunohistochemistry assessed ER, PR, HER2, stromal CD10 expression, and molecular subtypes. Nottingham Prognostic Index (NPI) was calculated for risk stratification.

**Results:**

Most patients were female, with predominance of T2 tumors (61.6%), IDC-NST (86.3%), and grade 2 tumors (79.5%). ER, PR, and HER2 positivity were observed in 52.1%, 37%, and 30.1% of cases. Basal-like and luminal B subtypes were most common (30.1% each). Stromal CD10 showed strong positivity in 49.3%, weak positivity in 30.1%, and negativity in 20.5%. CD10 expression showed a higher frequency among cases with T2 tumors, grade 2 histology, lymphovascular invasion, lymph node involvement, ER/PR negativity, HER2 positivity, and basal-like/HER2-enriched subtypes; however, these associations did not reach statistical significance.

**Conclusion:**

In low-resource settings, Stromal CD10 may have potential as an adjunct marker of tumor aggressiveness; however, in the present study, its associations with adverse clinicopathological parameters were not statistically significant. Larger multicentric studies with survival data are required for validation. Validation requires larger multicentric investigations with survival data.

## Introduction

In India, breast cancer represents a major national and international health burden and is the primary cause of cancer-related deaths among women. It surpassed lung cancer in 2020, accounting for 2.3 million new cases globally. By 2030, it is expected to account for 2 million cases.(1) Age, tumor size, histological grade/type, lymph node metastases, lymphovascular invasion, and hormone receptors (ER/PR/HER2) are examples of traditional prognostic variables.

Despite conventional signs (tumor size, grade, nodal status, ER/PR/HER2), the tumor microenvironment and heterogeneity make prognosis unpredictable, leading to high rates of metastasis and recurrence.(2) Zinc-dependent metalloproteinase, or stromal CD10, is a new prognostic marker.(3) CD10 was first thought to be a tumor-specific antigen; however, it has been discovered on a wide range of normal cellular types, including both hematopoietic and nonhematopoietic ones. Elevated stromal CD10, which is expressed in myoepithelial cells, neutrophils, and stromal fibroblasts, is associated with poor outcomes, including ER-negative status, high grade, nodal metastases, invasion, decreased survival, and poor response to HER2+ therapy. Myoepithelium/basement membrane loss facilitates the development of invasive cancer from ductal carcinoma in situ (DCIS).(4,5)

Because stromal cells are genetically stable, they offer therapeutic targets. Research highlights the relationship between stromal cells and tumor cells in the development and resistance to treatment.(6) In order to improve metastasis prediction and therapy stratification, this study evaluated CD10 immunohistochemistry in invasive breast cancer stroma and correlated expression with age, tumor size/grade/stage, lymph node status, and ER/PR/HER2.

To assess the significance of CD10 expression as a stromal marker in breast carcinoma and its correlation with histopathological and immunohistochemical parameters.

To determine the expression of CD10 as a stromal marker in breast carcinoma.

To correlate the stromal expression of CD10 with established immunohistochemistry parameters.

## Materials and Methods

**Fig. 1:**
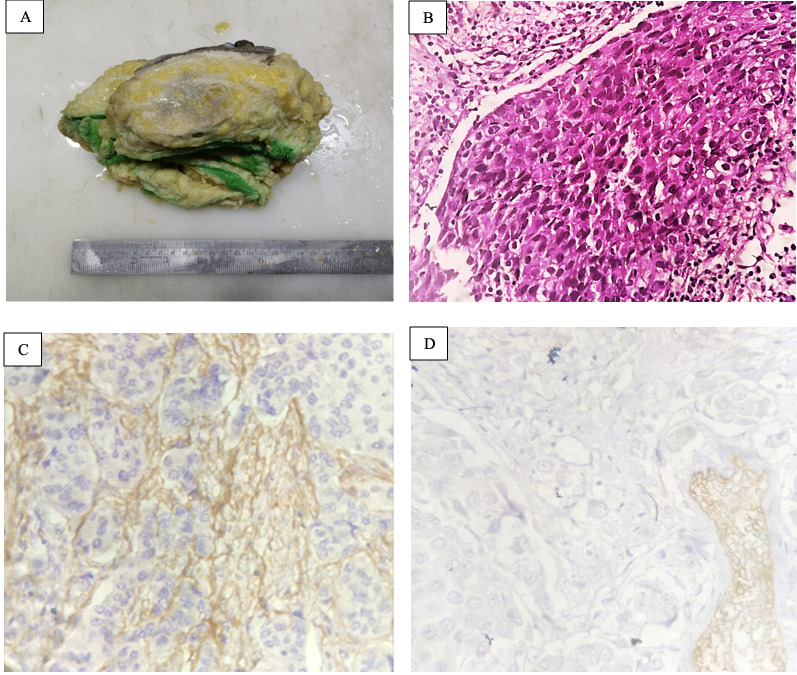
Gross and microscopic features of invasive breast carcinoma with stromal CD10 expression. (A) Gross specimen showing a 3.5 × 2.4 cm solid, pale-white tumor with irregular borders. (B) H&E section showing invasive breast carcinoma with moderate nuclear atypia, vesicular nuclei, and prominent nucleoli (H&E, ×40). (C) Strong stromal CD10 immunoexpression (IHC, ×40). (D) Weak stromal CD10 immunoexpression (IHC, ×40).

Seventy-three cases of invasive ductal carcinoma (IDC) of the breast were evaluated in this prospective study, which was carried out in the Department of Pathology in Medical College from April 2024 to December 2025 with ethical permission. All biopsy and mastectomy specimens diagnosed as invasive breast carcinoma during the study period were included; however, only cases with sufficient tissue for histopathological grading and complete immunohistochemical evaluation were analyzed; individuals who had previously received neoadjuvant chemotherapy or radiation were excluded. The samples were sectioned at 4 µm, fixed in 10% formalin, and subjected to gross examination (Fig. 1A). Two pathologists examined H&E slides to confirm the diagnosis, subtyped tumors, and graded IDC using the Nottingham modification of the Bloom-Richardson system (grade I: 3-5; II: 6-7; III: 8-9), assessing tubule formation (1: >75%; 2: 10%-75%; 3: <75%), nuclear pleomorphism (1: minimal; 2: moderate; 3: marked variation), and mitotic count (1: ≤11/10 hpf; 2: 12-23; 3: ≥23/10 hpf).

IHC was performed on 4 additional poly-L-lysine-coated sections for ER/PR (Allred/Quick Score: positive if ≥3, proportion 0-5 + intensity 0-3), HER2-neu (0-1+: negative; 2+: weak; 3+: strong overexpression), FISH was not done in HER2-neu equivocal cases, and CD10 (stromal cytoplasmic/membranous; 0: <10% negative; 1: 10%-30% weak; 2: >30% strong positive).

### Statistical Analysis

Statistical analysis was done using SPSS Info version 29. Categorical variables were summarized as frequency and percentage. Continuous variables were summarized as mean and standard deviation. Pearson chi-square test or Fisher exact test was used to compare interrelationships between the analyzed stromal expression of CD10 and clinicopathological variables. The results were considered statistically significant when the P value was < 0.05.

## Results

A total of 73 cases were analyzed, with the majority of patients in the 51–60 years age group (38.4%), followed by 41–50 years (28.8%). Females constituted almost all cases (98.6%). Most patients presented within less than 6 months of symptom onset (67.1%), and a family history was absent in 95.9% of cases. The distribution between left (50.7%) and right (49.3%) sides was nearly equal.

The tumor size was predominantly T2 (tumor greater than 20 mm but less than or equal to 50 mm) (61.6%), followed by T1 (tumor less than or equal to 20 mm) (21.9%) and T3 (tumor greater than 50 mm in greatest dimension) (16.4%). The most common tumor location was the upper outer quadrant (31.5%), followed by the upper inner quadrant (30.1%). Histopathologically, invasive ductal carcinoma (no special type) (Fig. 1B) was the most frequent diagnosis (86.3%), with other variants being rare. Most tumors had uninvolved margins (84.9%), were of histological grade 2 (79.5%), and were unifocal (84.9%).

Lymphovascular invasion was present in 58.9% of cases, while perineural invasion was rare (4.1%). Lymph node involvement was seen in 54.8% of patients. Ductal carcinoma in situ (DCIS) component was present in 60.3% of cases. Estrogen receptor positivity was observed in 52.1%, while progesterone receptor positivity was lower (37%). HER2 was positive in 30.1% of cases.

The molecular classification showed equal predominance of basal-like and luminal B subtypes (30.1% each), followed by luminal A (21.9%) and HER2-enriched (17.8%). CD10 expression was strongly positive (Fig. 1C) in 49.3% and weakly positive (Fig. 1D) in 30.1% of cases. According to the Nottingham Prognostic Index, most cases fell into the moderate (39.7%) and good (31.5%) prognostic categories, with fewer cases in excellent (20.5%) and poor (8.2%) groups (Table 1).

**Table 1:** Distribution of Breast Carcinoma

S.no	Category		Frequency	%
1	Age (Yrs)	31-40yrs	6	8.2
		41-50yrs	21	28.8
		51-60yrs	28	38.4
		61-70yrs	13	17.8
		71-80yrs	4	5.5
		81-90yrs	1	1.4
2	Tumor Size	T1 (Tumor less than or equal to 20 mm)	16	21.9
		T2 (Tumor greater than 20mm but less than or equal to 50mm)	45	61.6
		T3(Tumor greater than 50 mm in greatest dimension)	12	16.4
3	Histopathological diagnosis	Invasive Ductal carcinoma- no special type	63	86.3
		Mucinous carcinoma	2	2.7
		Invasive Breast Carcinoma with mucinous differentiation	1	1.4
		Invasive carcinoma with medullary like features.	1	1.4
		Invasive Ductal carcinoma- no special type with Mucinous carcinoma	1	1.4
		Invasive Ductal carcinoma-no special type-mixed features	1	1.4
		Invasive lobular carcinoma	1	1.4
		Invasive micropapillary carcinoma	1	1.4
		Invasive papillary carcinoma	1	1.4
		Invasive Solid papillary carcinoma	1	1.4
4	Histological Grade	Grade 1	6	8.2
		Grade 2	58	79.5
		Grade 3	9	12.3
5	Lymph Node Involvement	Absent	33	45.2
		Present	40	54.8
6	Estrogen Receptor	Negative	35	47.9
		Positive	38	52.1
7	Progesterone Receptor	Negative	46	63
		Positive	27	37
8	HER2	Equivocal	7	9.6
		Negative	44	60.3
		Positive	22	30.1
9	Molecular Classification	Basal like	22	30.1
		Her2 Enriched	13	17.8
		Luminal A	16	21.9
		Luminal B	22	30.1
10	Cluster of Differentiation 10	Negative	15	20.5
		Strong positive	36	49.3
		Weak positive	22	30.1
11	Nottingham Prognostic Index	Excellent	15	20.5
		Good	23	31.5
		Moderate	29	39.7
		Poor	6	8.2

The expression of CD10 showed varied distribution across clinicopathological parameters. Strong positivity was most common in the 51–60 years age group, while weak positivity was relatively higher in older age groups (61–70 years). Most tumors expressing CD10, irrespective of intensity, were T2 in size, with P value = 0.44. Histologically, the majority belonged to grade 2 tumors, with P value = 0.22 across all CD10 expression categories.

Lymphovascular invasion was more frequently seen in CD10-positive cases, with P value = 0.84, particularly those with weak positivity. Similarly, lymph node involvement was higher in CD10-positive groups (P value = 0.57), especially weakly positive and negative cases. Estrogen receptor expression was fairly balanced, though CD10-negative cases showed slightly higher ER positivity, with P value = 0.68. Progesterone receptor negativity predominated across all groups, with P value = 0.9.

HER2 negativity was most common irrespective of CD10 status, followed by HER2 positivity, with P value = 0.77. Molecular subtypes were fairly evenly distributed, with basal-like and luminal B subtypes being slightly more frequent among CD10-positive cases (P value = 0.93).

According to the Nottingham Prognostic Index, most cases across all CD10 categories fell into moderate and good prognostic groups, with fewer cases in excellent and poor categories, with P value = 0.93 (Table 2). Overall, CD10 positivity showed a non-significant trend toward adverse features such as lymphovascular invasion and nodal involvement.

**Table 2:** Association Between CD10 and Other Parameters

		CD10			Total
Strong positive	Weak positive	Negative
Age	31-40yrs	2(5.5)	4(18.1)	0	6
	41-50yrs	15(41.6)	4(18.1)	2(13.3)	21
	51-60yrs	17(47.2)	5(22.7)	6(40.0)	28
	61-70yrs	1(2.7)	7(31.8)	5(33.3)	13
	71-80yrs	1(2.7)	1(4.5)	2(13.3)	4
	81-90yrs	0	1(4.5)	0	1
Tumor Size	T1(<2cm)	10(27.7)	5(22.7)	1(6.6)	16
	T2(2-5cm)	20(55.5)	13(59.0)	12(80)	45
	T3(>5cm)	6(16.6)	4(18.1)	2(13.3)	12
Histological grade	1	1(2.7)	2(9.0)	3(20)	6
	2	31(86.1)	18(81.8)	9(60)	58
	3	4(11.1)	2(9.0)	3(20)	9
Lymphovascular invasion	Absent	10(45.4)	14(38.8)	6(40)	30
	Present	12(54.5)	22(61.1)	9(60)	43
Lymph node involvement	Absent	12(54.5)	15(41.6)	6(40)	33
	Present	10(45.4)	21(58.3)	9(60)	40
Estrogen Receptor	Negative	19(52.7)	10(45.4)	6(40)	35
	Positive	17(47.2)	12(54.5)	9(60)	38
Progesterone Receptor	Negative	23(63.8)	14(63.6)	9(60)	46
	Positive	13(36.1)	8(36.3)	6(40)	27
HER2	Equivocal	3(8.3)	3(13.6)	1(6.6)	7
	Negative	21(58.3)	12(54.5)	11(73.3)	44
	Positive	12(33.3)	7(31.8)	3(20)	22
Molecular classification	Basal like	11(30.5)	7(31.8)	4(26.6)	22
	Her2 Enriched	8(22.2)	3(13.6)	2(13.3)	13
	Luminal A	8(22.2)	4(18.1)	4(26.6)	16
	Luminal B	9(25.0)	8(36.3)	5(33.3)	22
NPI	Excellent	7(19.4)	6(27.2)	2(13.3)	15
	Good	12(33.3)	5(22.7)	6(40.0)	23
	Moderate	14(38.8)	9(40.9)	6(40.0)	29
	Poor	3(8.3)	2(9.0)	1(6.6)	6

## Discussion

The clinicopathological patterns observed in this prospective study of 73 cases of invasive ductal carcinoma are consistent with the epidemiology of breast cancer in India, where healthcare disparities, lifestyle changes, environmental exposures, and genetic factors cause the incidence to peak earlier (41–60 years, 67.2% combined; highest at 51–60 years, 38.4%) than in Western cohorts, necessitating targeted screening for middle-aged women(7,8). Despite female-focused programs, the predominantly female data (98.6%, 1 male case) are consistent with global/Indian statistics showing 0.4%–4.1% male occurrence due to hormonal/structural abnormalities(9,10).

According to Petrova et al, the majority (67.1%) showed symptoms before 6 months, although 32.9% did so after 6 to 12 months; Bachok et al reported intervals associated with severe disease(11,12). Family history was uncommon (4.1%), indicating that nongenetic reasons account for 5%–10% of the world's hereditary burden(13,14). According to Al Saad et al and Kim et al, laterality demonstrated near-equality (left, 50.7%; right, 49.3%), with no predictive influence(15,16).

The majority of tumors were T2 (61.6%), followed by T1 (21.9%) and T3 (16.4%), suggesting diagnostic delays despite screening potential. Larger sizes independently indicate poor survival/nodal distribution via aggressive proliferation(17). The central quadrant showed advanced stage/nodal risk (19.2%), while the upper quadrants showed higher glandular density (outer, 31.5%; inner, 30.1%; 61.6% combined)(18). IDC-NST predominated (86.3%), reflecting the 70%–80% incidence globally; mucinous (2.7%) and rare subtypes exhibit variability, with the latter offering a better prognosis through slower growth and less nodal involvement—highlighting the significance of precise histology for treatment(19,20). According to Nottingham criteria, the majority of intermediate-differentiated tumors that responded to prognostic grading were grade 2 (79.5%), grade 1 (8.2%), and grade 3 (12.3%)(21).

The multifocal instances (15.1%), which complicate surgery because of size/nodal/recurrence correlations, were dominated by unifocal cases (84.9%)(22,23). Clear margins (84.9%) reduced recurrence (compared with 15.1% positive), which was enhanced by >1 mm thresholds and methods such as shave margins(24,25). Common unfavorable characteristics from T2/grade 2 biology and referral bias were LVI (58.9%)(26), LN involvement (54.8%)(27), and DCIS (60.3%)(28,29); PNI was uncommon (4.1%)(30,31).

IHC revealed basal-like/luminal B (30.1% each), which were aggressive and recurrence-prone, luminal A (21.9%), HER2-enriched (17.8%)(32), ER+ (52.1%), PR+ (37%) lower in aggressive tumors signifying inadequate endocrine response(33), and HER2+ (30.1%)(34). The integration of size, grade, and nodes for survival prediction was verified by moderate NPI (39.7%), which is better than genomic assays in settings with low resources(35).

Stromal CD10 expression (strong positive, 49.3%; weak positive, 30.1%; negative, 20.5%) in this group of breast cancer patients was substantially associated with a number of prognostic factors. In contrast to nonsignificant age correlations in Sayed et al and Patil et al(36,37), CD10 positivity was more frequently observed in the 41–60-year age group; however, the manuscript does not demonstrate a statistically significant age-related association, indicating middle-aged Indian women as a high-risk cohort for aggressive illness through increased stromal remodeling. According to Sharma et al and Nabi et al(38,39), T2 tumors (2–5 cm; 55.5%) showed peak strong CD10 (P > 0.05), which is consistent with greater tumor size, grade, and nodal connections. This suggests intermediate-sized lesions with invasive potential and a worse prognosis. Despite nonsignificant trends in Praveena et al and Kamarudin et al(40,41), grade 2 cases showed 86.1% robust expression, underscoring CD10's function in intermediate-grade risk classification.

Strong CD10 was associated with LVI (61.1%)(42), LN involvement (58.3%), ER negativity (52.7%), PR negativity (63.8%), HER2 positivity (33.3%), basal-like/HER2-enriched subtypes (30.5%/22.2%), and moderate NPI (38.8%). These patterns make stromal CD10 a useful supplementary biomarker for detecting aggressive biology, improving prognosis beyond conventional markers, and suggesting more intensive treatment in settings with limited resources.

## Limitations

This was a single-center study with a lack of FISH and molecular correlation and did not evaluate additional stromal markers.

## Conclusion

Invasive breast carcinoma in this study predominantly affected middle-aged women and was mainly of invasive ductal carcinoma (NST), grade 2, and T2 stage. Stromal CD10 expression was common, with nearly half showing strong positivity. Although not statistically significant, CD10 positivity showed a trend toward adverse features such as lymphovascular invasion and lymph node involvement. Hormone receptor status showed moderate ER positivity, lower PR expression, and predominant HER2 negativity, with basal-like and luminal B subtypes being slightly more frequent. Overall, CD10 may serve as a potential adjunct marker of tumor aggressiveness, warranting further validation.

## Data Availability

The data that support the findings of this study are available from the corresponding author upon request.
